# Sleep Disruption and Cancer: Chicken or the Egg?

**DOI:** 10.3389/fnins.2022.856235

**Published:** 2022-05-19

**Authors:** Adrian Berisha, Kyle Shutkind, Jeremy C. Borniger

**Affiliations:** ^1^Cold Spring Harbor Laboratory, Cold Spring Harbor, NY, United States; ^2^Donald and Barbara Zucker School of Medicine at Hofstra/Northwell, Hempstead, NY, United States

**Keywords:** sleep disruption, cancer, anti-tumor immunity, stress, inflammation, HPA axis, sympathetic nervous system, hypocretin/orexin

## Abstract

Sleep is a nearly ubiquitous phenomenon across the phylogenetic tree, highlighting its essential role in ensuring fitness across evolutionary time. Consequently, chronic disruption of the duration, timing, or structure of sleep can cause widespread problems in multiple physiological systems, including those that regulate energy balance, immune function, and cognitive capacity, among others. Many, if not all these systems, become altered throughout the course of cancer initiation, growth, metastatic spread, treatment, and recurrence. Recent work has demonstrated how changes in sleep influence the development of chronic diseases, including cancer, in both humans and animal models. A common finding is that for some cancers (e.g., breast), chronic disruption of sleep/wake states prior to disease onset is associated with an increased risk for cancer development. Additionally, sleep disruption after cancer initiation is often associated with worse outcomes. Recently, evidence suggesting that cancer itself can affect neuronal circuits controlling sleep and wakefulness has accumulated. Patients with cancer often report difficulty falling asleep, difficulty staying asleep, and severe fatigue, during and even years after treatment. In addition to the psychological stress associated with cancer, cancer itself may alter sleep homeostasis through changes to host physiology and *via* currently undefined mechanisms. Moreover, cancer treatments (e.g., chemotherapy, radiation, hormonal, and surgical) may further worsen sleep problems through complex biological processes yet to be fully understood. This results in a “chicken or the egg” phenomenon, where it is unclear whether sleep disruption promotes cancer or cancer reciprocally disrupts sleep. This review will discuss existing evidence for both hypotheses and present a framework through which the interactions between sleep and cancer can be dissociated and causally investigated.

## Introduction

The importance of sleep has been recognized throughout history by the likes of the ancient Egyptians, the Greeks and the Romans-as demonstrated by the names of the gods they worshiped. Sleep is an essential component for maintaining normal physiology and re-establishing homeostasis. Sleep presents a period of vulnerability whereby the brain resides in a relative state of rest and displays reduced sensitivity to external stimuli (e.g., light, sound). Despite this period of vulnerability, sleep remains a highly conserved process whereby humans (and other primates) spend a significant portion of their lives asleep-about one-third of their total lives. Additionally, the amount of time spent asleep varies greatly in mammals, varying between 3 h and more than 20 h of sleep per day (Siegel, 2008). Indeed, long sleep is not a unique feature of primates as large animals also experience long sleep (Nicolau et al., 2000; Lesku et al., 2008). The functions of sleep remain to be fully elucidated, however, there are several prominent theories that have been postulated attempting to identify ultimate and proximate reasons for why we sleep (Benington and Craig Heller, 1995; Berger and Phillips, 1995; Walker and Stickgold, 2006; Siegel, 2009). In the last few decades, dozens of neuronal circuits have been mapped each contributing to the initiation, maintenance, and transition of sleep/wake states. Among these, subcortical neurons in the hypothalamus and brainstem are the most well-described. For example, hypocretin/orexin (HO) neurons are located within the lateral hypothalamus, a brain region that is essential for critical behaviors such as sleep, feeding, stress, and energy balance.

Normal human sleep consists of two major stages- non-rapid eye movement (NREM) and rapid-eye movement (REM) sleep. Most importantly, these different sleep stages can be easily distinguished using the electroencephalogram (EEG)-measured in microvolts (μV)-to detect changes in electric charge in the brain, in the form of neuronal oscillations or “brain waves.” EEG can be used to report the collective activity of many neurons recorded by electrodes placed on the surface of the scalp. These brain waves serve various physiological functions and correlate with the different behavioral states and physiological processes that occur during different sleep stages. In addition, electromyogram (EMG) biopotential signals can be used–in tandem with EEG–as an objective measurement for sleep characterization. Interestingly, scientists have discovered that these brain waves oscillate within specific frequency bands, ranging from very slow (<0.01 Hz) to ultra-fast (>1,000 Hz) oscillations (Penttonen et al., 1998). However, the raw EEG is usually described in terms of conventional frequency bands, including delta (δ; 0.5–4 Hz), theta (θ; 6–9 Hz), alpha (α; 9–12 Hz), sigma (ς; spindle band; 12–15 Hz), beta (β; 12–30 Hz), low (30–60 Hz), and high gamma (γ; 60–100 Hz) (Berger and Phillips, 1995). Consequently, the conventional bandwidth of clinical EEG focuses on the analysis of waveforms ranging from 0.5 to 70 Hz.

Non-rapid eye movement sleep, also known as slow wave sleep (SWS), is the first stage of sleep following the transition from wakefulness. NREM sleep constitutes about 75–80% of total time spent in sleep and is typically associated with reduced cortical neuronal activity. Hence, the most prominent brain waves in the EEG during NREM sleep are low frequency, high amplitude delta (δ) waves. These delta waves reflect synchronization between thalamocortical (TC) neurons-characterized by prolonged periods of hyperpolarization and increased membrane conductance. These changes at the cellular level are reflected at the circuit level as these periods of prolonged hyperpolarization result in a reduction in the encoding of afferent signals, thus temporarily depriving the cerebral cortex of external stimuli (Steriade, 2003). In humans, NREM sleep is subdivided into several stages numbered 1–3, each of which offers the relative depth of sleep and includes unique characteristics and changes in brain activity and physiology. The physiological changes that occur during NREM sleep include decreased blood pressure and heart rate as a result of decreased sympathetic-nerve activity, decreased ventilation and respiratory flow, decreased movements of the eyes under the closed eyelids, and significant changes in blood flow and metabolism (Nowak et al., 2021).

In contrast, REM sleep, also known as “paradoxical” or “active” sleep (as the EEG waveform closely resembles that of wakefulness) is the stage of sleep following the transition from NREM sleep. REM sleep constitutes the remaining 20–25% of total time spent in sleep and is typically associated with increased heart rate and respiration, fluctuations in brain/body temperature, increased brain activity, and active suppression of skeletal muscle activity apart from those controlling eye movement and inflation/contraction of the diaphragm. REM sleep is predominantly characterized by lower amplitude, higher frequency theta (θ) waves that reflect desynchronization of cortical neurons (Vertes, 1984; Frauscher et al., 2016). Theta oscillations are generated in the hippocampus where a combination of cellular characteristics and network interactions facilitate in the generation of rhythms that can be detected in the overlying cortex (O’Keefe, 1993; Colgin, 2013). Several studies have highlighted the role of pyramidal neurons and more recently, parvalbumin interneurons of the hippocampus in mediating theta frequency (Pike et al., 2000; Amilhon et al., 2015). REM sleep is the phase of sleep thought to be responsible for dreaming, although evidence suggests dreams may also occur during NREM sleep (Siclari et al., 2018). During REM sleep, there is total body voluntary muscle paralysis which is believed to be a mechanism that prevents us from acting out our dreams. This temporary paralysis of muscles is achieved through the inhibition of spinal motor neurons *via* brainstem GABA/glycinergic signaling (Carskadon and Dement, 2005; Brooks and Peever, 2012).

Our understanding of the interaction between sleep and circadian rhythms can be partially attributed to the two-process model of sleep regulation which has served as a major conceptual framework in sleep research for the last four decades. Today, we understand that sleep is regulated by a homeostatic, sleep-dependent process (Process S) that interacts with a sleep-independent circadian process (Process C) (Borbély et al., 2016). The two-process model of sleep regulation was organized based on the initial observation that variations in the sleep stages (i.e., sleep propensity) mirror the rhythm of our body temperature (Kleitman, 1933; Murray et al., 1958). Process S represents the sleep propensity, or sleep debt, which increases during wakefulness and declines during sleep. Process C, the circadian component of sleep is closely related to the circadian rhythms of metabolic and endocrine processes, indicating an important role of sleep in maintaining proper energy homeostasis, growth and development (Huang et al., 2011; Depner et al., 2014). Desynchronization of normal circadian rhythms impairs physiological functions in disease processes including metabolic diseases, cardiovascular diseases, and cancer (Fu and Lee, 2003; Sahar and Sassone-Corsi, 2009; Arble et al., 2010; Savvidis and Koutsilieris, 2012; Chaves et al., 2019). In fact, in 2007, the International Agency for Research on Cancer (IARC) classified “shift work that involves circadian disruption” as a probable human carcinogen (Group 2A in the IARC classification of *Known and Probable Human Carcinogens*). The synchronization of circadian rhythms is an essential component to sleep homeostasis, as the regulation of sleep/wake cycles are mainly controlled by daily circadian rhythms. In this review, we will discuss how sleep disruption may promote tumorigenesis through alterations in a variety of physiological processes such as energy balance and immunity. Reciprocally, we will discuss how cancer-mediated alterations in our physiological processes may lead to aberrant neural activity, affecting a myriad of behavioral changes including disruption of sleep/wake cycles.

## Sleep Disruption-Induced Changes to Systemic Physiology

The prevalence of sleep disorders in modern societies is increasing, affecting 20–40% of the general population (Chattu et al., 2019), likely in part due to increased shift work and exposure to artificial light at night. Sleep disturbances and disruptions in circadian synchronization are associated with the development of several disease states including cancer (Fu and Lee, 2003; Savvidis and Koutsilieris, 2012). Sleep disruption is common across different types of cancers, with the highest prevalence experienced by patients with breast cancer (Davidson et al., 2002; Budhrani et al., 2015). Although a consensus definition of “sleep disruption” does not exist, approximately 30–70% of cancer patients report sleep problems before and during treatment, which is about two times higher than in the general population (Savard et al., 2001; Ancoli-Israel et al., 2006; Palesh et al., 2010; Fiorentino et al., 2011). The most common problems related to sleep in breast cancer are insufficient sleep, hypersomnia, sleep fragmentation, poor sleep efficiency (i.e., time spent asleep/time in bed), hot flashes, and circadian misalignment. To examine the relationship between sleep and cancer risk, studies have largely focused on sleep duration, presence of sleep disorders, and night shift work as risk factors driving cancer.

A growing body of research links sleep disruption and circadian misalignment to subsequent tumor initiation and growth (Hansen, 2001; Schernhammer et al., 2006; Van Dycke et al., 2015). Given that the overall prevalence of sleep disorders is on the rise (Ferrie et al., 2011; Chattu et al., 2019), it is necessary to assess its contribution to tumorigenesis. Notably, a prospective study of approximately 24,000 women by Kakizaki et al. (2008) demonstrated an inverse association between sleep duration and risk of breast cancer, where shorter sleep duration (i.e., 6 h or less) was associated with an increased risk of breast cancer. The hazard ratio (HR) of women who slept less than 6 h was 1.62, of those who slept 8 h was 1.14 and of those who slept 9 h or more was 0.72. In addition, the deleterious effects of sleep disruption on systemic physiology has also been explored in animal models, where several studies implicate sleep disruption in both tumor progression and cancer-related mortality (Maragno-Correa et al., 2013; Hakim et al., 2014). In this section, we will delve into inflammation, altered metabolism, anti-tumor immunity, stress, and sympathetic nervous system activity in driving tumor initiation, growth, and subsequent metastasis as potential mechanisms linking disrupted sleep to cancer outcomes.

### Sleep Disruption and Anti-Tumor Immunity

Sleep and the immune system engage in a form of bi-directional communication, and comprise two essential components in health and disease (Lorton et al., 2006). As we will discuss in later sections of this review, activation of the immune system (e.g., *via* immune-cell-derived cytokines) alters sleep, and reciprocally, sleep affects both the innate and adaptive arms of the immune system. Several human studies have demonstrated sleep disruption-induced elevations in circulating immune cells, including granulocytes (e.g., neutrophils) T cells, and B cells (Born et al., 1997; Faraut et al., 2011; Besedovsky et al., 2016), indicative of an inflammatory state. In addition, studies in mice have recently illuminated the role of sleep deprivation in promoting tumor progression as a result of impaired anti-tumor immunity (De Lorenzo et al., 2018; Huang et al., 2021). A study by De Lorenzo et al. (2018) demonstrated the deleterious effects of 18-h sleep deprivation on antitumor immunity in a murine model of melanoma. The results demonstrated that sleep deprivation reduced the number of cytotoxic cells (i.e., NK and CD8+ T cells), concurrent with increased numbers of pro-tumor regulatory T cells, in the tumor microenvironment (TME). In addition, similar effects were observed systemically as the number of CD4+ and CD8+ T cells were reduced in the blood of sleep-deprived mice along with a decreased population of dendritic cells (antigen-presenting), indicative of an immunosuppressive phenotype. The effects of this immunosuppressive phenotype were exemplified by an earlier onset of lung metastasis in sleep-deprived animals, leading to increased metastatic burden. Previous work by De Lorenzo et al. (2015) demonstrates a role of the sympathetic nervous system (SNS) in sleep disruption-induced reductions in the number and cytotoxic activity of NK cells, as treatment with the beta-2 adrenergic receptor (β_2_-AR) antagonist propranolol reversed the effects of sleep disruption on NK cell phenotypes. However, the causality of sleep disruption-induced alterations to the immune landscape and subsequent tumor growth remains unclear.

Until recently, most studies investigating the immune landscape in the context of sleep deprivation used flow cytometric analysis with little-to-no use of high-dimensional single-cell techniques. These approaches are required to garner critical information on the molecular and cellular interactions underpinning sleep disruption associated malignancies. A study by Liu et al. (2021) provide unique insight into the dynamic single-cell alterations underlying sleep disruption-induced rewiring of the immune cell landscape. Blood was collected from six healthy individuals before and after 24-h sleep deprivation (preSU and postSU, respectively) and then subsequently analyzed using mass cytometry by time of flight (CyTOF) and single-cell RNA sequencing (scRNA-seq). Coincident with the aforementioned studies, Liu et al. (2021) found increases in circulating T cells (TC) as well as decreases in myeloid cells (MYE) postSU as assessed by classical lineage markers using scRNA-seq. Next, the five major immune cell lineages (TC, NK, BC, MC, and DC) were further sub-clustered into transcriptionally distinct subsets. Following 24-h sleep deprivation, single-cell clustering identified the onset of lymphocythemia (i.e., increased number of lymphocytes) as demonstrated by increases in CD8^+^ effector memory TCs (CD8 T_EM_), proliferating TCs (mitotic TC, T-mito), and exhausted TCs (Tex) (as a percent of CD45+). Next, Liu et al. (2021) sought to identify the molecular events associated with 24-h sleep deprivation which was achieved through the analysis of differentially expressed genes from blood immune cells in the postSU group compared with the preSU group. All six participants in the study showed an increase in several inflammatory genes including markers of DNA damage, AP-1 family genes (*JUN*, *FOS*), IFNG and interferon-related developmental regulator 1 (*IFRD1*). In addition, gene ontology (GO) identified the upregulated genes across participants postSU were enriched in the AP-1 pathway, leukocyte activation, and cellular responses to stress (i.e., cellular senescence). The most prominent downregulated genes postSU were those involved in metal ion homeostasis and detoxification. These findings indicate that sleep deprivation induces general oxidative stress and an inflammatory state in circulating immune cells. Importantly, TCs, BCs, and DCs were the cell types most strongly affected by sleep deprivation among individuals according to their upregulated genes. Within CD4^+^ TCs, Liu et al. (2021) demonstrated that sleep-deprivation resulted in an increase Th17 differentiation markers CCR6, CXCR3, the cell proliferating marker Ki67, and apoptotic marker CD279. In addition, increased levels of CXCR3, CCR6, and the autoimmune-related BC (ABC) marker T-bet were identified in B cells after sleep-deprivation, indicative of autoimmune-associated changes in effector lymphocytes postSU.

**FIGURE 1:**
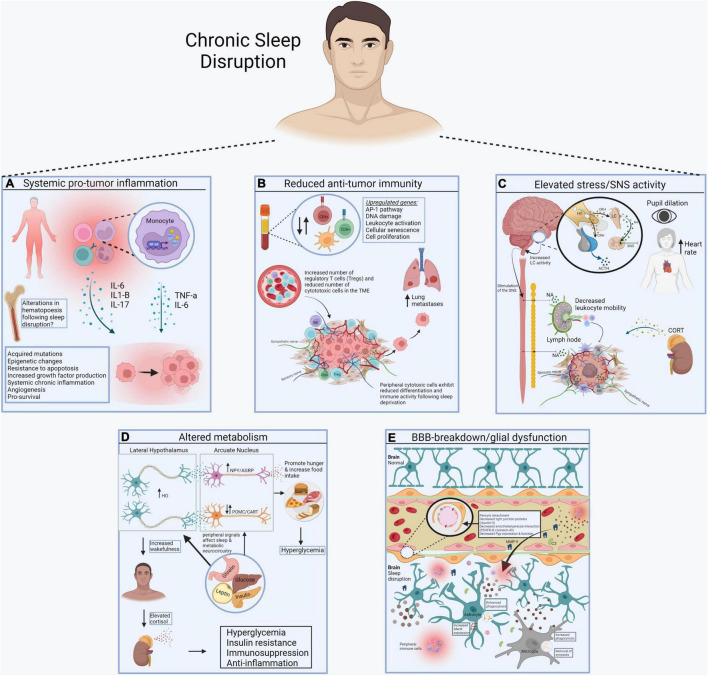
Systemic consequences of sleep disruption. **(A)** Sleep disruption-induced inflammation establishes a pro-tumor inflammatory environment in peripheral tissues, including the increase in proinflammatory cytokines like IL-6, IL1-b, and IL-17, mediated by the transcription factor NF-kB. **(B)** Sleep disruption reduces anti-tumor immunity. Alterations in the number of circulating and intra-tumor lymphocytes and myeloid cells contributes to immunosuppression in cancer. Reduced differentiation of cytotoxic cells further prevents cancer elimination. **(C)** Sleep disruption engages the HPA axis and autonomic nervous system. Glucocorticoids and catecholamines have widespread effects on the immune system and energy balance important for tumor progression. **(D)** Sleep disruption alters systemic metabolism. Wake-promoting neurons (e.g., hypocretin/orexin) regulate the activity of others that control food intake and metabolic health (e.g., POMC, AgRP, NPY neurons). **(E)** Sleep disruption promotes the breakdown of the blood brain barrier (BBB). Disrupted sleep results in vascular endothelial cell dysfunction and inflammation, further contributing to BBB impairment. This allows inflammatory molecules in blood to reach the brain, where they alter the function of sleep/wake regulatory systems (Made with BioRender.com).

Importantly, Liu et al. (2021) revealed that functional marker expression of cytotoxic cells (i.e., NK and CD8^+^ TCs) were altered postSU, including decreased expression of transcription factors (e.g., T-bet) that normally promote differentiation and functional polarization of cytotoxic cells. In addition, sleep-deprivation upregulated levels of *PFN1*, a negative regulator of cytotoxic cell killing and migratory functions. Thus, the cytotoxic cells present in postSU blood show transcriptional alterations that favor an increase in an inflammatory phenotype and coincident decrease in cytotoxic activity. Lastly, Liu et al. (2021) demonstrated that the particular cell-cell interactions in the blood after sleep-deprivation were those mainly involved in inflammatory activation of lymphocytes to other cells and chemotaxis of MYEs to other cells. In addition, unique intercellular interactions between TCs, NKs, and BCs were identified with upregulated expression of the ephrin family and their receptor EPH family in the postSU group which are implicated in the onset of inflammation and disease pathogenesis. Thus, sleep deprivation promotes an inflammatory environment in peripheral tissues with reduced differentiation and immune activity of cytotoxic cells, likely increasing susceptibility to tumorigenesis. The findings by Liu et al. (2021) can be used to inform the cellular and molecular mechanisms underlying sleep disruption-induced pathogenesis, including its potential contribution to development and progression of cancer (Figure 1).

### Sleep Disruption-Induced Inflammation

Inflammation is an evolutionarily ancient process wherein cells of the innate and adaptive arms of the immune system are activated and recruited to sites of host insult or pathogen invasion. Equally important to host defense is inflammation’s role in tissue repair and regeneration. Although vital for normal health, several decades of research have firmly implicated inflammation in the development and progression of cancer. Site specific chronic inflammation and subsequent cancer development is a common theme for many organ systems. Prominent examples highlighting this relationship include cigarette smoking and chronic viral hepatitis increasing the risk of lung cancer and liver cancer, respectively. Epidemiologic evidence also demonstrates that inhibition of inflammation with NSAIDs reduces incidence and mortality of many cancers following long term use (Rothwell et al., 2011).

Substantial experimental evidence demonstrates that sleep disruption promotes inflammation in both animal models and in human studies. In one study, rats that were selectively sleep deprived of REM sleep for 72 h showed significant increases in proinflammatory cytokines compared to controls. The elevated markers include IL-1 alpha, IL-1 beta, IL-6, IL-17, TNF-alpha, corticosterone, and homocysteine. The shift to a pro-inflammatory state persisted for at least 1 week, as levels of IL-17, TNF-alpha, corticosterone, and homocysteine remained elevated despite having the opportunity for normal sleep and sleep rebound (Yehuda et al., 2009). Even just one night of sleep loss in healthy adults induces an inflammatory response. In a study where volunteers underwent partial sleep deprivation (awake from 11 p.m. to 3 a.m.), morning monocyte production of IL-6 and TNF-alpha was significantly elevated compared to prior mornings following uninterrupted sleep (Irwin et al., 2006). The rise in proinflammatory signaling following sleep loss is largely mediated through the transcription factor NF-kB. Following a single night of partial sleep deprivation, mononuclear cell NF-kB was significantly elevated compared to uninterrupted or recovery sleep. NF-kB is intimately tied to chronic inflammation and tumorigenesis where it provides cells with resistance to apoptotic insults and leads to the production of growth factors (Karin and Greten, 2005). In another study, 24 healthy adults undergoing one night of partial sleep deprivation similarly showed greater expression of IL-6 and TNF-alpha relative to baseline. These inflammatory cytokines were accompanied by increased monocytic expression of activated (phosphorylated) STAT1 and STAT5 (Irwin et al., 2015). STAT proteins transduce signals to the nucleus where they function as transcription factors, with certain STAT proteins (STAT1) acting to increase anti-tumor immunity and others (namely STAT3) facilitating cancer-promoting inflammation (Yu et al., 2009). Interestingly, there appears to be sex differences in sleep disruption driven inflammation. In the morning after sleep loss, LPS-stimulated IL-6, and TNF-alpha concentrations were equally elevated in both females and males. However, production of these cytokines remained elevated in females into the evening whereas it decreased in males (Irwin et al., 2010). These findings suggest sleep loss may exert a differential risk for inflammatory driven disorders across sexes.

The evidence linking sleep disruption and induction of a pro-inflammatory state is vast, however, the underlying mechanisms remain to be fully studied. Sleep induced inflammation is driven by complex neuro-immune interactions, likely mediated through neuroendocrine axes (e.g., HPA axis) and the autonomic nervous system, as previously discussed. As discussed above, sleep disturbance activates the HPA axis. In turn, a chronically active HPA axis can lead to glucocorticoid resistance of immune cells, wherein immune cells lose sensitivity to the anti-inflammatory effects of glucocorticoids (Webster et al., 2001). A natural decrease in sympathetic nervous system (SNS) activity that occurs during the night is also prevented when sleep loss occurs. This increased sympathetic outflow is carried forward into the day and influences inflammation. Noradrenergic signaling through beta receptors can activate NF-kB and induce the production of inflammatory cytokines (Irwin and Cole, 2011). In a study examining sleep and cardiovascular disease, mice undergoing chronic sleep fragmentation developed larger atherosclerotic plaques and produced less hypocretin in the lateral hypothalamus compared to controls. Additionally, the aortas and blood of sleep deprived mice contained higher levels of monocytes, neutrophils, and macrophages. Hypothalamic hypocretin reduced gradually and was inversely correlated with leukocytosis throughout the sleep disruption paradigm. It was determined that the link between reduced hypocretin and leukocytosis is mediated through hypocretin sensitive pre-neutrophils, whose production of colony stimulating factor-1 is decreased in response to hypocretin. Taken altogether, hypocretin dysfunction alters the immune landscape, leading to a relative leukocytosis that favors atherosclerosis. Whether alterations in hematopoiesis in response to sleep disruption similarly increases tumorigenesis is yet to be determined.

Inflammation’s role in tumor initiation is multifactorial. Sleep induced inflammation is systemic and differs from the traditional model of “site specific” chronic inflammation leading to organ-specific tumors. Cytokine and immune cell alterations following sleep disruption may create an environment favoring cancer development and progression. For example, mice that constitutively produce IL-15, a proinflammatory cytokine and growth factor, developed fatal lymphocytic leukemia (Fehniger et al., 2001). Macrophage-migration inhibitory factor, another proinflammatory cytokine, suppresses the activity of p53 (Hudson et al., 1999). IL-1 is required for angiogenesis and invasiveness as mice deficient in IL-1alpha or IL-1beta exhibit impaired tumor development and blood vessel growth in melanoma, mammary adenocarcinoma, and prostate cancer (Voronov et al., 2003). CSF-1 serves as a regulator of mammary tumor metastasis as its overexpression accelerated the progression and invasion of the primary tumor to other sites (Lin et al., 2001). Inflammation can trigger mutagenesis through ROS formation by macrophages and neutrophils (Canli et al., 2017). Inflammatory cytokines such as IL-1, IL-6, and TNF-alpha can activate epigenetic machinery in epithelial cells, altering expression of oncogenes and tumor suppressor genes (Grivennikov, 2013). Inflammation has also been shown to induce tumor initiating stem-cell like cells from normal epithelium (Schwitalla et al., 2013). Further, cytokine receptor signaling through NF-kB, JAK/STAT, and other cascades may induce pro-survival pathways, increasing the likelihood for a cancerous cell to survive and produce successful clones (Grivennikov et al., 2009). Thus, unresolved inflammation that arises due to sleep disruption sustains a proinflammatory environment, both locally within tissues and systemically. This favors tumorigenesis through aberrant cytokine signaling and subsequent cell growth, increased cell turnover, and immune evasion.

### Sleep Disruption, Stress, and the Sympathetic Nervous System

Psychological or psychosocial stress has been implicated in the etiology of several prominent diseases in humans including clinical depression, cardiovascular disease (CVD), human immunodeficiency virus (HIV/AIDS), and cancer (Cohen et al., 2007). Moreover, chronic stress has emerged as a key factor associated with cancer initiation, progression, and subsequent metastasis in animal models and humans (Sklar and Anisman, 1981; Kim-Fuchs et al., 2014; Le et al., 2016). A combination of retrospective, prospective, and observational studies have explored the effects of psychological stress on tumorigenesis, revealing that stressful life events (e.g., death of a family member, divorce, etc.) frequently precede the appearance of several forms of malignancies including breast cancer (Cooper et al., 1989; Geyer, 1991; Ginsberg et al., 1996). Several behavioral changes that occur as adaptations or coping responses to stressful life events such as loss of sleep and exercise, increased smoking or alcohol consumption, and reduced adherence to medical regimens constitute several pathways by which stressors increase susceptibility to developing a chronic disease. In addition, two stressor-induced endocrine responses provide additional pathways influencing disease risk; namely the hypothalamic-pituitary-adrenocortical (HPA) axis and the sympathetic-adrenal-medullary (SAM) system. Glucocorticoids, the primary output of the HPA axis, regulate a multitude of physiological processes (e.g., glucose mobilization, immunosuppression) and are also involved in resetting the circadian clock in peripheral tissues (Balsalobre et al., 2000; Dibner et al., 2010). The prolonged activation of these endocrine responses can interfere with their control of normal physiological systems including immune, metabolic, and neurological functions, resulting in increased susceptibility to the development of physiological and psychological disorders.

Sleep disruption triggers a stress response, which in turn increases concentrations of adrenal glucocorticoids and epinephrine (i.e., adrenaline). Research findings have firmly established that our own life-experiences, such as stressful events, have an impact on the quality, duration and physiology of sleep. Reciprocally, sleep influences stress, resulting in an overlap between stress and sleep disruption-induced physiological changes. Thus, it is difficult to disentangle the independent effects of sleep disruption alone, as sleep disruption results in emotional and physiological stress with large implications for subsequent health and disease. The connection between sleep and stress has recently been described as Li et al. (2020) demonstrated that hypocretin/orexin (HO) to CRH neuron signaling causes stress-induced insomnia.

Since the reports of the discovery of the HO neuropeptides and their receptors in 1998, research has firmly established the primary role of HO neurons in the maintenance of wakefulness (Sakurai, 2005, 2007; Adamantidis et al., 2007; Inutsuka and Yamanaka, 2013; Tyree et al., 2018). HO neurons are sensitive to circulating peripheral signals such as acyl-ghrelin, leptin, and glucose (Yamanaka et al., 2003; Adamantidis and De Lecea, 2009) which become deregulated throughout the course of cancer progression. In response, HO neurons alter their firing rates to elicit appropriate physiological responses to putatively re-establish homeostasis. HO neurons are more active during wakefulness (e.g., during sleep deprivation), resulting in an increase in the activity of post synaptic neurons in brain regions that receive their projections. More specifically, there are two critical efferent projections from HO neurons that are likely responsible for changes in peripheral physiology observed in cancer: the HPA axis and autonomic output nuclei [e.g., locus coeruleus (LC), ventrolateral medulla, A5, A1]. HO neurons project to various autonomic output nuclei in the brainstem, namely the locus coeruleus, which projects to the spinal cord to alter peripheral physiology *via* the sympathetic nervous system (SNS) (Geerling et al., 2003; Samuels and Szabadi, 2008a,b). The physiological changes that accompany increased activity of noradrenergic neurons in the locus coeruleus is increased arousal and vigilance and increased activity of sympathetic nerves in the periphery as assessed by dilation of the pupils (Murphy et al., 2014; Costa and Rudebeck, 2016; Joshi et al., 2016; Liu et al., 2017). Importantly, sympathetic nerves are implicated in exacerbating primary tumor growth and subsequent metastasis in animal models of breast cancer and in human patients (Sloan et al., 2010; Monje et al., 2020; Zahalka and Frenette, 2020), providing an anatomical pathway linking stress, sleep disruption, and cancer in the body.

A study by Kamiya et al. (2019) demonstrated that sympathetic innervation of tumors accelerates progression of human breast cancer xenografts in mice. Sympathetic nerves within the TME were constitutively activated, which was achieved using adeno-associated virus (AAV) delivery of a mutant sodium channel that remains open, promoting tonic depolarization of neuronal membranes. Two-photon calcium imaging of sympathetic nerve endings in the TME confirmed stimulation of sympathetic nerves, which was concurrent with increased tumor volume of the primary tumor in the mammary fat pad and increased metastasis to the lungs, a primary site of breast cancer metastasis. However, AAV delivery of diphtheria toxin A subunit (DTA) to ablate innervating sympathetic nerves, resulted in decreased primary tumor growth with no measurable metastasis. The results of the DTA-induced elimination of sympathetic nerves were recapitulated in animal models that experienced increased stimulation of parasympathetic nerves in the TME, thus highlighting the duality between the two divisions of the autonomic nervous system relevant to breast cancer (Kamiya et al., 2019). Importantly, Kamiya et al. (2019) demonstrated a similar phenomenon in human breast tumors of 29 patients who underwent surgical resection of primary breast tumors. Of these 29 patients, 10 patients subsequently experienced recurrence of breast cancer whereas the remaining 19 patients did not. Immunofluorescence staining of surgically resected primary tumors revealed a positive correlation between sympathetic nerve fiber densities and cancer recurrence. Specifically increased sympathetic nerve densities were observed in the primary tumors of patients who subsequently experienced recurrence, which was also associated with a lower recurrence-free survival rate. The opposite correlation was observed for parasympathetic nerve densities. Thus, increased stimulation of sympathetic nerves (or reduced parasympathetic input) within the TME results in enhanced tumor growth, progression, subsequent metastasis, and increased incidence of recurrence in animal models and patients with breast cancer. Mechanistically, this may have to do with the actions of the sympathetic nervous system on local immune cells important for anti-tumor immunity. Kamiya et al. (2019) also demonstrated that genetic sympathetic nerve denervation (and parasympathetic neurostimulation) reduced the expression of immune checkpoint molecules (e.g., PD-1, PD-L1)-exploited by cancer cells in order to evade the host immune-response- in the TME in animal models of breast cancer. Once again, these findings highlight the effects of local sympathetic nerve output on anti-tumor immunity and subsequent cancer progression. In addition, sympathetic nerves were closely associated with PD-1+ and FOXP3+ tumor infiltrating lymphocytes (TILs) with innervation of PD-L1+ tumor tissue in human breast cancer (Kamiya et al., 2019). Activation of the SNS decreases leukocyte (i.e., CD4+ and CD8+ T cells) mobility in peripheral tissues, as systemic administration of the SNS neurotransmitter NA and/or administration of the βAR agonist isoprenaline sequestered leukocytes in lymph nodes (Devi et al., 2021). Devi et al. (2021) demonstrated this impaired immune response was due to SNS-induced vasoconstriction and subsequent hypoxia which resulted in increased calcium signaling within leukocytes, ultimately reducing their mobility. Altogether, these results indicate a critical role for the SNS in driving tumor progression and promoting an immunosuppressive, pro-tumorigenic environment characterized by increased neurotransmitter signaling, hypoxia and reduced leukocyte motility.

In addition to the control HO neurons exert on autonomic nuclei and subsequent SNS activity in the periphery, HO neurons also modulate activity of the HPA axis. Both intracerebroventricular (icv) administration of HO and optogenetic stimulation of HO neurons results in a rapid elevation of circulating glucocorticoids (e.g., corticosterone, cortisol), the primary humoral output of the HPA axis (Kuru et al., 2000; Bonnavion et al., 2015). In agreement with the results from preclinical models, sleep deprivation in humans activates the HPA axis, as sleep deprived individuals demonstrate amplified cortisol levels (Minkel et al., 2014). In addition, sleep deprivation results in a marked increase in corticotrophin-releasing hormone (CRH) expression and release into various brain regions in rats (Fadda and Fratta, 1997). The activation of the HPA axis and subsequent secretion of glucocorticoids by the adrenal cortex is highly relevant to tumorigenesis and tumor progression as glucocorticoids are widely recognized for their anti-inflammatory, immunosuppressive effects that promote tumor initiation and progression (Barnes, 1998; Coutinho and Chapman, 2011). In addition to the systemic effects, stressors elicit activation of the LC through the actions of CRH, a key component of the HPA axis (McCall et al., 2015). The net effect of CRH binding to their receptors (CRHRs) in the LC is increased neuronal discharge resulting in high-tonic activity and increased activation of the SNS in the periphery (Samuels and Szabadi, 2008a,b; Valentino and Van Bockstaele, 2008). Thus, it is likely that sleep deprivation (partially due to increased hypocretin neuronal activity) has both direct and indirect influences on sympathetic output. Direct (projections to the LC) and indirect (engagement of the HPA-axis) actions of hypocretin neurons on LC activity results in increased stimulation of the sympathetic nervous system.

In addition to the interplay between these two brain regions controlling stress and SNS activity in the central nervous system (CNS), stress and SNS activity are intimately linked in the periphery. Treatment of spleen-derived NK cells with the glucocorticoid receptor agonist dexamethasone upregulates expression of β_2_-adrenergic receptors (β_2_-AR) on NK cells, suggesting that glucocorticoids are able to induce expression of β_2_-AR (De Lorenzo et al., 2015). This results in a decrease in the number and cytotoxic activity of NK cells, further highlighting the dynamic interplay between sleep disruption-induced stress, SNS activity, and immune-suppression relevant to cancer. Thus, the consequence of these CNS interactions extends to the periphery, further promoting the propensity of cancer cells to grow and proliferate in an immunosuppressive environment.

### Sleep Disruption-Induced Metabolic Alterations

Mounting evidence relates sleep loss to the development of obesity, diabetes, and other metabolic abnormalities. Metabolic abnormalities because of poor sleep are likely a risk factor for the development of many cancers. Interactions between sleep and metabolic regulation is primarily mediated through the hypothalamus. In a similar fashion to sleep, hypothalamic control over metabolism occurs through two networks that inhibit each other. Neuropeptide Y (NPY) and agouti-related protein (AgRP) neurons promote hunger and actively inhibit pro-opiomelanocortin (POMC) and amphetamine-related transcript (CART) neurons, which suppress appetite. Further, both populations of neurons are sensitive to leptin and ghrelin, which serve as major humoral cues of hunger and energy expenditure. Leptin simultaneously suppresses activity of NPY and AgRP neurons and activates POMC and CART neurons whereas ghrelin has the inverse effect in both sets of neurons (Rolls et al., 2010).

In addition to the actions of peripheral hormones on neurocircuitry, increased risk of metabolic dysfunction may be linked to sleep-induced alterations of HO neurons. Interactions between HO neurons and hypothalamic metabolic centers likely contribute to many of the metabolic abnormalities seen in those who experience chronic sleep disruption. HO neurons can sense and respond to glucose, leptin, and ghrelin, allowing for adaptive augmentation of arousal in response to changes in energy balance (Yamanaka et al., 2003; Tyree et al., 2018; Walker and Borniger, 2019). Further supporting the role of HO neurons in metabolism is the anatomical connectivity between HO neurons and metabolic nuclei of the hypothalamus. HO axons directly contact NPY and POMC neurons in the arcuate nucleus of rats and signaling through these axons has been shown to regulate their activity in a manner reciprocal to leptin (Muroya et al., 2004). Moreover, ghrelin-induced food intake is significantly reduced in mice pretreated with antibodies against HO and HO knockout mice (So et al., 2018). In the context of sleep deprivation, the activity of HO neurons increases to maintain wakefulness against increased pressure to sleep (Wu et al., 2002). Thus, sleep loss resulting in overactivity within HO neurons likely plays a role in the metabolic effects of sleep loss as these same neurons influence the activity of metabolic centers in a way that promotes eating and energy mobilization.

Low-grade systemic inflammation due to obesity and hyperglycemia can increase the risk and progression of many cancers, including malignancy of the breast (Greten and Grivennikov, 2019). Furthermore, liver, pancreatic, colorectal, and breast cancer all show an association with Type II diabetes mellitus (Vigneri et al., 2009; Suh and Kim, 2011). In one study, patients with breast cancer had increased glucose concentrations during the time of their diagnosis when compared to age-matched controls (Ryu et al., 2014, p. 201). Similarly, in a study assessing the effects of multiple nights of partial sleep loss, it was found that the rate of glucose clearance following injection was reduced by 40% after sleep restriction compared to sleep recovery nights in a sample of healthy volunteers aged 18–27 years (Spiegel et al., 1999). Glucose effectiveness, which is the ability of glucose to mobilize itself independent of an insulin response, and acute insulin response to glucose were both found to be 30% lower during mornings after sleep loss compared to mornings after sleep recovery. Sleep loss was also accompanied by an increase in afternoon and evening plasma cortisol levels. The authors proposed that the raised cortisol concentrations later in the day reflect impaired negative-feedback control of the HPA axis, which is a component of age-related insulin resistance. This study was one of the first to show that just several days of sleep disruption in healthy people is linked to drastic alterations in metabolic function, and sleep loss induced changes that mimic characteristics of aging (Spiegel et al., 1999)-which is the greatest risk factor for almost all cancers.

It is well-known that chronic hyperglycemia has profound negative health consequences, in part mediated through the formation of advanced glycation end products (AGEs). AGEs form throughout life *via* non-enzymatic glycation of various proteins and lipids, a process that is accelerated by hyperglycemia and inflammation. Interactions between AGEs and its receptor, receptor for advanced glycation end products (RAGEs), alters vascular homeostasis and contributes to the development and progression of cardiovascular disease (Senatus and Schmidt, 2017). AGE-RAGE interactions and downstream signaling may also be involved in tumorigenesis. The actions of AGEs associated with cancer development include activation of RAGE leading to signaling cascades favoring proliferation, inflammation, and increased levels of ROS (Lin et al., 2016). AGEs have been implicated in the promotion of several tumor types *in vitro*, including breast, colon, and lung cancer, among others (Takino et al., 2010; Ishibashi et al., 2013; Chen et al., 2014). Epidemiologic and animal studies support these findings with high-AGE diets increasing the risk of many cancer types (Shimomoto et al., 2012; Foster et al., 2014; Jiao et al., 2015). In summary, sleep disruption alters systemic metabolism in a way that favors tumor initiation and growth.

## Sleep Disruption-Induced Changes to the CNS

The blood brain barrier (BBB) is a complex organization of cerebral endothelial cells, pericytes, and supporting astrocytes which serves to provide a stable environment for neural function through regulation of peripheral neurotransmitter infiltration, hormones, macromolecules, and the ionic microenvironment around synapses and axons. Moreover, the BBB allows for stringent control of CNS homeostasis by monitoring and detecting circulating toxins, pathogens, and inflammation. Hence, the BBB limits paracellular permeability due to the presence of tight junctions between the continuous monolayer of endothelium in the brain vasculature. However, the presence of nutrient transporters (e.g., glucose, amino acids, ketones) and receptors (e.g., for insulin, leptin) on endothelial cells lining the cerebral vasculature enables the delivery (*via* passive diffusion, active transport or receptor-mediated endocytosis) of essential molecules required for neural development and proper neural function. Thus, the unique structure, function, and location of the BBB enables this structure to serve as key regulator of entry into the CNS- serving a crucial role in the protection of the brain parenchyma from injury and disease.

### Sleep Disruption-Induced Blood Brain Barrier Disruption

Studies have illuminated the role of sleep in providing a “restorative function” in that sleep promotes removal of neurotoxic waste products that accumulate in the interstitial space during wakefulness *via* a “garbage collector” system termed the glymphatic system (i.e., cerebrospinal fluid transport) (Xie et al., 2013; Ding et al., 2016; Hauglund et al., 2020). In addition, recent studies have demonstrated the role of both endogenous circadian rhythms and sleep in promoting the clearance of metabolites along the BBB at night (Cuddapah et al., 2019). This is mainly achieved *via* regulation of BBB permeability through the activity of permeability-glycoprotein multidrug transporters [also known as Pgp, multidrug resistance protein 1, or ATP-binding cassette sub-family B member 1(ABCB1)] which are less active at night (i.e., reduced function), thus increasing permeability of the brain overnight. The presence and activity of Pgp on brain capillary endothelial cells serves as an efflux pump to expel substrates back into the circulation after they initially diffuse into the endothelial cells, restricting entry into the brain parenchyma. Additional studies have demonstrated the role of sleep loss in impairment of BBB function and subsequent increase in BBB permeability to proinflammatory cytokines (e.g., TNF-a) and immune cells (He et al., 2014a,b; Opp et al., 2015; Hurtado-Alvarado et al., 2017; Medina-Flores et al., 2020). A study by He et al. (2014b) investigated whether chronic sleep restriction (CSR) contributed to pathophysiological processes in the brain. Indeed, they demonstrated that CSR affects genes involved in vascular endothelial function and inflammation. In addition, they demonstrated that CSR increased the uptake of sodium fluorescein 10 min after intravenous injection in the brainstem, cerebellum, and subcortical regions, demonstrating a functional breakdown in the barrier. Similarly, Medina-Flores et al. (2020) described the interactions between brain endothelial cells and pericytes that promote BBB disruption after sleep loss in rats. They demonstrated that daily 20-h sleep restriction for 10 days [i.e., chronic sleep restriction (CSR)] reduces pericyte-brain endothelial cell interactions as assessed by decreased expression of the pericyte-endothelial cell interaction markers connexin 43 and platelet-derived growth factor receptor-B (PDGFR-B) in the cerebral cortex and hippocampus. In addition, CSR promotes brain pericyte detachment–which was not concurrent with apoptosis of pericytes–from the capillary wall in both the cerebral cortex and hippocampus, resulting in impairment of blood brain barrier function as assessed by subsequent increase in permeability to sodium fluorescein and Evans Blue.

In alignment with these findings, previous studies in rats have demonstrated that co-culture of endothelial cells and pericytes are more effective in increasing transendothelial electrical resistance (TEER) and lowering permeability to both low and large molecular weight tracers as compared to monocultures of endothelial cells alone (Nakagawa et al., 2009). In addition, Medina-Flores et al. (2020) demonstrated that disruption of endothelial cell-pericyte interactions (i.e., BBB impairment) was also associated with decreased expression of the tight junction protein claudin-5 in both the cerebral cortex and hippocampus which was associated with overexpression of matrix metalloproteinase-9 (MMP-9). Lastly, Medina-Flores et al. (2020) demonstrated that the BBB disruption in CSR rats was concurrent with an increased blood-brain barrier permeability to the chemical compound rhodamine 123, which is often used as a functional reporter of PGP activity. Thus, these findings highlight the contribution of sleep loss, independent of tumor burden, on BBB disfunction. Given the contribution of various disease states (e.g., cardiovascular disease, kidney disease, diabetes, cancer) in promoting sleep disruption, it is becoming increasingly necessary to further characterize the relationship between sleep and pathological conditions. In addition, further studies need to assess the potential contribution of sleep loss-induced BBB disruption in promoting an additional, unregulated route by which peripheral signals enter the brain parenchyma in various pathologies, exacerbating aberrant neural circuitry in a vicious cycle.

### Sleep Disruption and Glial Cells

**FIGURE 2:**
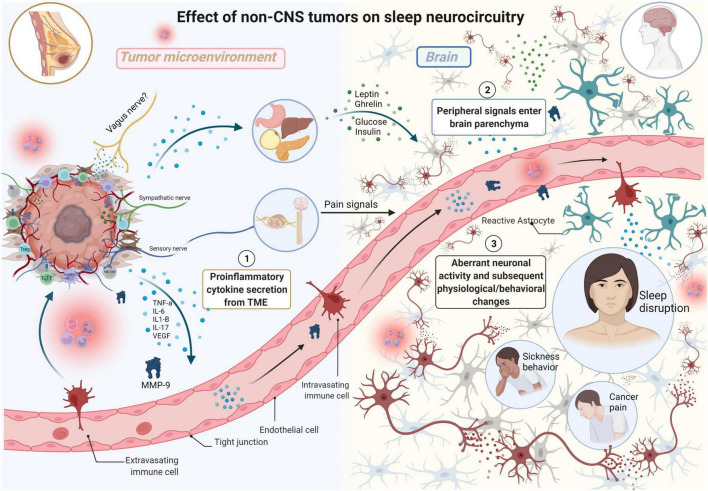
The effects of non-CNS tumors on sleep neurocircuitry. TME-derived proinflammatory signals (e.g., TNFa, IL-6, IL1-B, IL-17) enter systemic circulation, eventually moving into the brain parenchyma impacting neurocircuitry including sleep. TME-derived proinflammatory signals also exert effects on distant organs including stomach, liver, pancreas, and adipose tissue resulting in elevated secretion of satiety/hunger hormones (e.g., leptin, ghrelin) and glucose and insulin which all exert differential effects on sleep neurocircuitry in the CNS. Sensory nerves in the TME relay information from the TME, resulting in the production of severe pain in various malignancies including breast cancer. This results in neurochemical changes in the spinal cord and forebrain, including elevated levels of noradrenaline (NA), in the brain parenchyma with negative consequences on both quality of life and survival. Aberrant neuronal activity results in subsequent physiological/behavioral changes including sleep disruption, sickness behavior, and cancer pain (Made with BioRender.com).

Glial cells are present in both the central and peripheral nervous systems and serve many vital functions (e.g., myelin production) and are essential regulators in the formation, maintenance, and function of synapses. The major type of glial cells in the central nervous system (i.e., brain and spinal cord) are astrocytes, oligodendrocytes, microglia, and ependymal cells–each of which contain their respective function and molecular/cellular characteristics. Neurons and glial cells occupy a comparable amount of space in nervous tissue; however, the number of glial cells outnumber that of neurons. Thus, a comprehensive review of the effects of sleep disruption on tumorigenesis–and reciprocally, the effects of peripheral tumors in promoting sleep disruption–must encompass the potential contributions of glial cells. Recently, a role of glial cells in the regulation of sleep-wake cycles is emerging, highlighting the dynamic interactions present within neural circuits that influence sleep in both health and disease (Garofalo et al., 2020; Ingiosi et al., 2020). For example, a study by Bellesi et al. (2017) demonstrated that sleep loss enhances astrocyte phagocytosis as observed by an increase in the number of synaptic elements (e.g., spine head, axon, dendrite) surrounded by peripheral astrocytic processes (PAPs) in mice that underwent sleep deprivation (SD) or chronic sleep restriction (CSR). In addition, translating ribosome affinity purification technology and microarrays identified increased astrocytic *Mertk* expression- a “wake” gene upregulated in both spontaneous wake and sleep deprivation. In addition, Bellesi et al. (2017) demonstrated that only CSR is associated with microglia activation in the mouse cerebral cortex, assessed by analyzing the morphology (i.e., branching) of microglial cells since it correlates closely with their state of activation. Subsequently, the group demonstrated that there was higher expression of C3, a major component of the innate immune complement cascade required for microglial phagocytosis, in SD and CSR mice. Thus, CSR is associated with microglial activation and increased phagocytosis without a notable increase of inflammatory mediators in the CSF. The observed glial phagocytosis may serve different functions such as the removal of abundant synapses that become established during extended wake periods. In addition, studies have demonstrated that microglial activation can occur in response to systemic inflammation and, in turn, can communicate systemic inflammation to the brain [91]. Thus, systemic inflammation induced by sleep deprivation, cancer, or chemotherapy treatment can result in glial-mediated synaptic elimination which may contribute to the cognitive impairment observed in cancer patients and/or represent a mechanism by which sleep deprivation affects cognitive function. In addition, aberrations in neuronal circuitry during sleep deprivation that promote systemic inflammation and subsequent tumorigenesis may be attributed to synaptic remodeling by activated glial cells (Figure 2).

## Non-CNS Tumor Effects on Sleep Neurocircuitry

Poor sleep experienced by cancer patients and survivors could be attributed to the presence of one or more underlying sleep disorders. Thus, one of the most prominent concerns in cancer patients is the onset of sleep disorders including difficulty falling asleep, problems maintaining sleep, poor sleep efficiency and early awakening. Early immunologists treated the nervous and immune systems as largely separate entities. However, today there is consensus that there is bi-directional communication between the nervous and immune systems in both health and disease states. To date, there are several pathways that have been proposed in the field of neuroimmunology as being important for the communication between the immune system and central nervous system: the neural route (e.g., vagal afferent signaling), BBB active transport of cytokines and secretions from BBB cells, passive diffusion at circumventricular organs which lack a BBB (e.g., median eminence), infiltration of peripheral immune cells, as well as interactions with meningeal lymphatics (Quan and Banks, 2007; Kipnis, 2016; Prinz and Priller, 2017). Thus, peripheral cues can signal to the CNS whereby the CNS coordinates appropriate responses (i.e., sickness behavior) as an adaptive strategy to an immune challenge. For example, intravenous (IV) administration of inflammatory cytokines interleukin-1 (IL-1), tumor necrosis factor alpha (TNF-alpha), and interleukin-6 (IL-6)- all of which are upregulated during cancer progression- has been shown to induce sickness behavior (including sleep disruption) similar to those observed during infection in both mice and humans (Focà et al., 1983; Lesnikov et al., 1991; Ching et al., 2007). Interestingly, two additional reflex responses to systemic LPS have been well-studied, including both fever and the activation of the HPA axis (Hosoi et al., 2000; Romanovsky, 2005). Given the role of peripheral signals in influencing the CNS, it is unsurprising that LPS as well as additional proinflammatory cytokines (e.g., IL1-B and TNFa) exert somnogenic effects, in that they are involved in the regulation of sleep (Opp, 2005). In addition, studies have demonstrated that LPS-induced lethargy is mediated by alterations to HO activity (Grossberg et al., 2011). In this section we will discuss additional routes (e.g., humoral, neural) that are critical for relaying information to inform the brain about the peripheral environment.

### Humoral Route

Tumors in the periphery present a systemic challenge, altering metabolic, immune, and (likely) cognitive capacity. The nervous system and peripheral tumors (i.e., non-CNS tumors) engage in bidirectional communication as well. A growing body of literature illuminates the role of nerves in cancer initiation, progression, and subsequent metastasis through direct interactions with cancer cells or through interactions with stromal cells in the TME (Faulkner et al., 2019; Monje et al., 2020; Zahalka and Frenette, 2020). Just as normal, healthy tissues recruit and maintain innervation of the peripheral nervous system (PNS) (e.g., autonomic nervous system) to promote regeneration and repair of tissue, peripheral tumors co-opt these pathways to aid in the recruitment of nerves into the tumor microenvironment through the release of neurotrophic factors (e.g., NGF, BDNF, NT-3). Subsequent outgrowth of nerves into the TME results in enhanced cholinergic or adrenergic signaling in the TME, resulting in tumorigenesis and increased aggressiveness of gastric and breast cancer, respectively (Sloan et al., 2010; Hayakawa et al., 2017). In addition, innervation of sensory nerves in the TME mediates pain responses relevant to cancer progression. Consequently, increasing tumor burden in peripheral tissues has been shown to affect neural activity in the brain, with significant consequences on sleep-controlling neurocircuitry in the hypothalamus (Walker and Borniger, 2019). The hypothalamus is sensitive to peripheral signals and is able to sense these signals (*via* humoral and neural routes) and subsequently alter neural activity (Francis and Borniger, 2021). Consequently, the hypothalamus is able to generate physiological and behavioral responses through its influence on both the autonomic nervous system (i.e., sympathetic and parasympathetic divisions) and the hypothalamus-pituitary-adrenal (HPA) axis. A more thorough review on the role of the hypothalamus as a systemic integrator in both homeostasis and in response to homeostatic challenges (e.g., cancer) can be found here (Francis and Borniger, 2021).

Borniger et al. (2018) demonstrated tumor-induced sleep and metabolic abnormalities in a mouse model of non-metastatic breast cancer, independent of behavioral deficits or cachexia. The metabolic abnormalities were reflected in alterations to satiety hormone signaling (i.e., leptin and ghrelin) and hepatic glucose processing which was found to coincide with peripheral IL-6-driven inflammation. IL-6 levels were elevated both in the tumors and serum of tumor-bearing mice which was also associated with increased protein concentrations of the IL-6 regulated transcription factor pSTAT3 and increased expression of downstream targets of IL-6 signaling in the liver (e.g., *Stat3*). In addition, brain and muscle activity were detected using EEG and EMG, respectively, to assess changes to sleep-wake states throughout cancer progression. Interestingly, the presence of the peripheral tumor was found to disrupt sleep-wake states in the later course of tumor progression as evidenced by reduced time spent awake and an increase and fragmentation of NREM but not REM sleep. These observed sleep-wake aberrations were mainly attributed to enhanced activity of HO neurons as evidenced by increased cFos immunoreactivity during the active phase in tumor-bearing mice. Importantly, the altered sleep patterns were not due to altered immune activation in the brain, as there were no indications of increased pro-inflammatory cytokines (IL-6, TNF-a, IL1-B) in several brain regions. Borniger et al. (2018) also demonstrated that the use of a HO-receptor antagonist (Almorexant) and not neutralizing antibodies against IL-6, attenuated tumor-induced impairments in glucose metabolism and improved sleep quality–indicative of active sensing of HO neurons to peripheral signals on metabolic and immune status, as previously reported (Adamantidis and De Lecea, 2009). In addition, the reported alterations in metabolism (i.e., impaired glucose tolerance, spontaneous hyperglycemia) were attributed to HO-mediated control of the sympathetic nervous system. Chemical sympathectomy (*via* the neurotoxin 6-OHDA) attenuated metabolic abnormalities in tumor-bearing mice as evidenced by restoration of blood glucose concentrations and normalization of several hepatic genes involved in gluconeogenesis/glycolysis (ldha, gck, pklr). These findings provide mechanistic insight into tumor-driven alterations in sleep-wake neurocircuitry that may be coupled to changes in metabolism or immune signaling in the periphery. These findings warrant further discussion on reprogramming the use of current clinically approved hypocretin receptor antagonists (e.g., Suvorexant) for improving metabolic and sleep aberrations in cancer patients (Walker and Borniger, 2019).

### Neural Route (*via* Sensory Neurons)

Sensory neurons form the afferent division of the peripheral nervous system and are primarily responsible for conveying various signals arising from the viscera and the skin to the central nervous system, in turn activating neuroendocrine and visceromotor reflexes. The cell bodies of sensory neurons reside in the dorsal root ganglia (DRG) of the spinal cord or along cranial nerves. Sensory neurons are subdivided into visceral and somatic sensory neurons, which transmit sensory information primarily from internal organs and skin and skeletal muscles, respectively. In addition, sensory neurons contain different receptors for different stimuli (e.g., thermoreceptors, mechanoreceptors, nociceptors, photoreceptors, and chemoreceptors) which in turn allow for the perception of various sensations including pain, temperature, and touch.

Recent studies highlight the role of sensory neurons in the initiation, migration, progression and metastasis of pancreatic and breast cancer (Demir et al., 2015; Saloman et al., 2016; Le et al., 2021). In addition, sensory neurons are implicated in the production of severe pain in breast, prostate, colon, pancreatic, and bone cancer, which is in part due to perineural invasion of cancer cells (Cain et al., 2001; Liebig et al., 2009; Andersen and Kehlet, 2011; Mantyh, 2013). Cancer pain does not only influence quality of life but also affects the survival of cancer patients (Mantyh, 2006). Nociceptors (i.e., pain receptors) are densely packed on afferent fibers of sensory neurons where they primarily relay noxious stimuli to the spinal cord that are then, *via* ascending pathways, conveyed to various brain regions to elicit pain sensations. Nociceptors are capable of detecting different forms of noxious stimuli (ATP, IL1, IL6, NGF, VEGF, TNFa, protons) that are secreted by cancer cells and other components of the TME (Mantyh et al., 2002). Consequently, sensory neurons alter their pattern of expression of various signaling molecules which partly underlies increased sensitization and subsequent hyperalgesia/allodynia. In addition to the changes in sensory neurons, the spinal cord and forebrain both undergo neurochemical and structural changes as chronic pain develops during cancer progression which alters neuronal activity (Honore et al., 2000a,b). A key brain region that has been extensively studied in several pain conditions and is involved in the modulation of pain is the locus coeruleus (LC), through the release of NA and subsequent action on adrenergic receptors (Brightwell and Taylor, 2009; Llorca-Torralba et al., 2016; Taylor and Westlund, 2017). Importantly, the LC has direct relevance to sleep as the LC is important for promoting arousal (Foote et al., 1983; Berridge and Waterhouse, 2003; Aston-Jones and Cohen, 2005). Neurons in the LC fire tonically at 1–3 Hz during wakefulness, fire less during NREM sleep and are essentially silent during REM sleep (G. Aston-Jones and Bloom, 1981a,b). When a painful stimulus is applied at the periphery, both ascending and descending pain pathways are activated, in which the LC is a key structure in both pathways (Hwang et al., 2001; Howorth et al., 2009). In the ascending pathway, the pain information is first transmitted to the spinal cord and subsequently along ascending axons to supraspinal structures, including the paragigantocellular nucleus (PGi), which exerts excitatory effects on the LC (Ennis et al., 1992). After reaching the LC, pain information is transmitted to other brain regions such as the amygdala, hypothalamus, thalamus, and cortex engaging more complex behaviors in response to pain (e.g., sleep and stress-related responses). Additionally, given the critical role of LC-noradrenergic neurons in the transition between sleep and wakefulness, studies have shed some insight into the possible involvement of the LC in pain-related sleep disruption (Koh et al., 2015). Since the hypocretin-mediated sleep-wake transition is heavily dependent on its projections to LC noradrenergic neurons, there may also be an important functional role of HO neurons in nociceptive perception and subsequent sleep-wake regulation in response to pain (Hagan et al., 1999; Mohammad Ahmadi Soleimani et al., 2015; Mohammad-Pour Kargar et al., 2015; Ahmadi-Soleimani et al., 2020). Thus, sensory neurons play a critical role in relaying pain information (e.g., tumor-induced inflammation) arising from the periphery to the CNS with impacts on sleep-controlling brain regions such as the lateral hypothalamus and LC (i.e., increased wakefulness). In addition, sleep disruption can increase pain sensitivity, and pain can in turn disrupt sleep physiology (Alexandre et al., 2017).

On the other hand, the descending noradrenergic pathway involves those mainly projecting to the spinal cord (Howorth et al., 2009). Interestingly, the descending noradrenergic pathway from the LC to the spinal cord is mainly ipsilateral, although there is also crossing over of the information at the midline to innervate the opposite side of the dorsal horn. However, these studies demonstrate the projections of the pain-responsive noradrenergic neurons originating in the LC to the lumbar dorsal horn. Whether or not a subset of pain-responsive noradrenergic neurons originating in the LC and projecting to other divisions of the spinal cord has not been assessed. The lateral horn of the spinal cord contains the neuronal cell bodies of the sympathetic division. Thus, it is possible that pain-induced stimulation of the LC results in the activation of descending pathways that project to the lateral horn of the spinal cord, resulting in the activation of the SNS and further promoting tumor progression. Consequently, growing tumor burden in the periphery increases pain perception *via* ascending pathways that project to the LC. As a result, noradrenergic neurons in the LC increase their firing rates, resulting in downstream effects on peripheral tissues–establishing a vicious cycle.

### Active Surveillance of the Periphery by the Vagus Nerve Cranial Nerve X (CNX)

The discovery of the cholinergic anti-inflammatory pathway provides another example of the important function of peripheral nerves in sensing, encoding, and relaying inputs to the CNS regarding our body’s internal state (e.g., fluctuations in peripheral cytokines and toxins, tissue injury) (Tracey, 2002). Since the characterization of the cholinergic anti-inflammatory pathway, a plethora of studies have established the role of local and systemic inflammation in the activation of vagal efferent fibers, resulting in suppression of cytokine release from macrophages (Tracey, 2007). Most of the evidence for the action of the anti-inflammatory pathway have been demonstrated in a model of LPS administration in rodents (Borovikova et al., 2000a,b; Wang et al., 2003). Unsurprisingly, the vagus nerve may have a critical role in informing the brain about the tumor microenvironment with subsequent consequences on neuronal activity (Gidron et al., 2005).

Interestingly, several studies highlight a contribution of the vagus nerve in the sleep-promoting effects of IL1-B. A study by Hansen and Krueger (1997) demonstrated that subdiaphragmatic vagotomy blocks the sleep and fever-promoting effects of IL1-B in rats. Rodents were separated into two groups, those that received a vagotomy (Vx) or sham surgery (i.e., vagus nerve remained intact). Subsequently, both Vx and sham groups were given an intraperitoneal (IP) injection of low-dose (0.1 μg/kg) IL-1B and the amount of time spent in sleep was analyzed. Hansen et al. observed that administration of low-dose IL1-B in sham rats increased NREM sleep, whereas administration of low-dose IL1-B in Vx rats failed to induce significant changes in NREM sleep compared to controls. In addition, Hansen et al. demonstrated that the administration of low-dose IL1-B in sham rats induced a significant increase in body temperature (i.e., fever response), whereas the increase in body temperature was completely blocked in Vx animals. One important aspect of this study is that vagotomy did not block the sleep and fever responses when rats were subject to high-dose (2.5 μg/kg) IL1-B. Thus, subdiaphragmatic vagotomy completely abolishes low-dose IL1-B-induced NREM sleep and fever responses. However, the inability of the vagotomy to block the sleep and fever responses in rats subjected to high-dose of IL-1B can be attributed to mechanisms that are not dependent on intact subdiaphragmal vagi. These findings indicate that in addition to the subdiaphragmatic vagus control of sleep and fever in response to acute inflammation, alternative pathways exist that influence the CNS at more severe levels of inflammation (i.e., chronic inflammation or sepsis). Thus, elevation of IL1-B in the systemic circulation (induced by sleep disruption, cancer, chemotherapy, etc.) effect sleep neurocircuitry *via* vagal input and likely enter the brain parenchyma *via* aforementioned mechanisms (i.e., BBB-mediated transport and/or passive diffusion at circumventricular organs) resulting in aberrant neuronal activity.

### Systemic Inflammation and Blood Brain Barrier Breakdown

Sleep is regulated by both humoral and neuronal mechanisms that are dependent on each other. Several studies have demonstrated that proinflammatory cytokines (e.g., IL-1B and TNF-a) have a somnogenic effect, increasing both sleep (e.g., NREM) and lethargy following peripheral immune activation (Opp, 2005). Interestingly, the concentrations of TNF-a and IL-1B in the CNS display circadian oscillations, with IL-1B and TNF-a mRNA expression and protein content in the brain coinciding with the amount of NREM sleep (Bredow et al., 1997; Taishi et al., 1997). In addition, many immunomodulators of sleep-wake behaviors including cytokines, chemokines, and growth factors, all of which may become deregulated in cancer patients (Dranoff, 2004). Alterations in the levels of these signaling molecules have dynamic effects on many biological processes including release of neurotransmitters, peptides, and hormone secretions which have profound effects on sleep-wake neurocircuitry.

**FIGURE 3:**
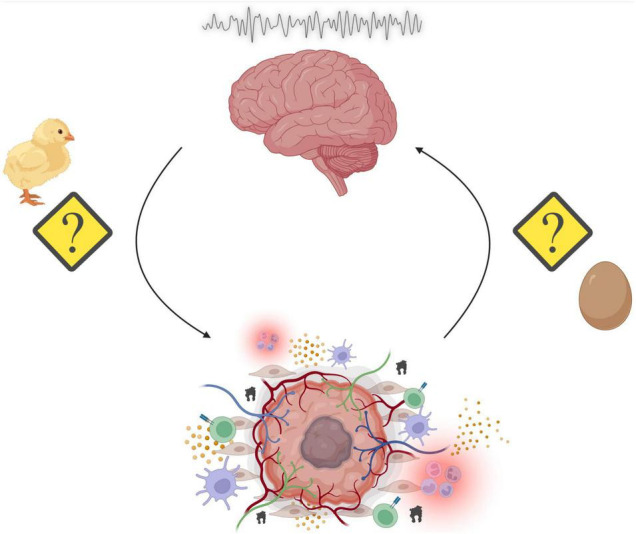
Summary of the overarching principles highlighted in this review. Sleep disruption may promote tumorigenesis through alterations in a variety of physiological processes such as energy balance, immunity, and stress. Reciprocally, the genesis of peripheral tumors and the subsequent assembly of the tumor microenvironment (TME) results in cancer-mediated alterations to our physiological processes. The consequences include aberrant neural activity, which affect a multitude of behavioral outputs including disruption of sleep/wake cycles. This results in a “chicken or the egg” phenomenon, where it is unclear whether sleep disruption promotes cancer or cancer reciprocally disrupts sleep (Made with BioRender.com).

In addition to the established somnogenic effects of proinflammatory cytokines and chemokines (Opp, 2005), *via* vagal afferents (Hansen and Krueger, 1997) and additional mechanisms, proinflammatory cytokines have also been implicated in disruption and impairment of the BBB. A key component of the BBB architecture is the presence of tight junction proteins between brain capillary endothelial cells that serve to limit the movement of substances into the brain. Numerous studies have demonstrated a role of systemic inflammation (e.g., cancer) and cancer therapies (e.g., chemotherapy) in cytokine-mediated breakdown of the BBB [reviewed in Wardill et al. (2016)]. In addition, more recent studies have demonstrated that the exposure of the endothelium to immune cell-derived proinflammatory cytokines (e.g., TNF-alpha, IL-1b, IFN-g) results in disruption of BBB integrity and enhanced leukocyte endothelial adhesion and migration. Thus, it is becoming increasingly important to recognize the contribution of BBB disruption as an additional route by which aberrant neuronal activity arises during cancer progression. In addition, tumor-induced disruption of the BBB promotes host death in preclinical models (Kim et al., 2021). A study by Kebir et al. (2007) demonstrated a role of human T_H_17 lymphocytes in promoting blood-brain barrier disruption and central nervous system inflammation. Interestingly, T_H_17 lymphocytes were able to migrate across the BBB both *in vitro* (using human BBB-EC’s) and *in vivo* as analyzed by human CNS postmortem tissues from individuals with multiple sclerosis, a disease with well-characterized BBB disruption. Kebir et al. also demonstrated that the binding of both IL-17 and IL-22, two identified cytokine products of T_H_17 lymphocytes, to their receptors (IL-17R and IL-22R, respectively) on human brain endothelium was critical for the increased permeability of the BBB. IL-17 consists of a family of proinflammatory cytokines secreted primarily by activated T-helper type 17 (T_H_17) lymphocytes. IL-17 plays an important role in the homeostasis of tissues in health but also contributes to autoimmunity, chronic inflammation and invasion in various inflammatory diseases such as multiple sclerosis, rheumatoid arthritis, inflammatory bowel diseases and type I diabetes (Jin and Dong, 2013). A role for IL-17 in promoting breast cancer progression and metastasis has been described, which is associated with a worse prognosis (Du et al., 2012; Chen et al., 2013; Jin and Dong, 2013, p. 1; Coffelt et al., 2015). Within the tumor microenvironment, IL-17 is primarily secreted by tumor-infiltrating lymphocytes in two murine models of breast cancer (Du et al., 2012). Thus, IL-17 production by immune cells in the TME both promotes breast cancer progression and results in disruption of the BBB. The consequences of BBB disruption are increased entry of proinflammatory cytokines into the brain parenchyma, resulting in an additional route of entry by which somnogenic cytokines alter sleep neurocircuitry. This, in turn, alters the activity of the HPA axis and SNS signaling in the periphery, further promoting cancer progression (Figure 3).

## Clinical Implications

### Clinical Data-Sleep Disruption and Breast Cancer

Although sleep disruption because of cancer is a well-established symptom of malignancy, its significance has been largely overlooked in traditional oncology treatment regimens. In one study, about 50% of women with non-metastatic breast cancer reported symptoms of insomnia prior to surgery, whereas rates on insomnia in men diagnosed with prostate cancer was about 30% prior to surgery (Savard et al., 2011). Clinical studies suggest that sleep disruption is particularly relevant in breast cancer. In addition to being highly prevalent, sleep problems are independently associated with a higher risk of earlier death and poor treatment response (Palesh et al., 2014; Innominato et al., 2015). Disrupted sleep patterns in breast cancer are also evidenced by alterations in the rhythmic secretion of cortisol, which is regulated by the central circadian clock in the suprachiasmatic nuclei (SCN) (Buijs et al., 2003; Abercrombie et al., 2004; Kiessling et al., 2017). VIP (vasoactive intestinal peptide) neurons within the SCN rely on tightly coordinated clock gene and neuronal rhythms to control their input to PVN corticotrophin-releasing hormone producing neurons (Jones et al., 2021). Normally, cortisol levels build up throughout the night, reach peak levels around the time of waking, and then steadily decline through the day (Weitzman et al., 1971). Studies have found that aberrant cortisol rhythms can even serve as a predictor of breast, lung, and ovarian cancer mortality, where patients with “flat” or abnormal rhythms show earlier mortality (Sephton et al., 2000, 2013; Schrepf et al., 2015). Experimental studies exploring the mechanistic link between circadian and cortisol rhythm disruption and cancer outcomes may be able to transform this relationship from a prognostic factor to the focus of treatments.

### Epidemiological Data and Controversy in the Field

As with many emerging fields, the current epidemiological evidence relating sleep quality, sleep duration, and shift work to subsequent cancer development is controversial. Several epidemiological studies have found that women working night shifts have a significantly elevated risk of breast cancer (Manouchehri et al., 2021). Similar relationships were observed for prostate and gastrointestinal tract cancer (Schernhammer et al., 2003; Kubo et al., 2006). However, several recent systematic reviews and meta-analyses have reported no associations between altered sleep and cancer initiation (Fritschi et al., 2013; Li et al., 2015; Chen et al., 2018). In addition, a recent paper by Titova et al. (2021) employed the use of mendelian randomization (MR), an epidemiologic technique used to determine the causal role of genetic variants for disease risk, to assess the effect of sleep duration on cancer risk. Titova et al. (2021) concluded that the MR study showed a casual association between both short and long sleep duration and risk of some site-specific cancers but not overall cancer. Titova et al. (2021) concluded that there is a lack of robust evidence to support causal associations of sleep duration with risk of overall and site-specific cancers. However, recent prospective studies demonstrate an increased risk of cancer in men who reported sleep duration of 5–6 h per night compared with those who slept 7–8 h (Gu et al., 2016). Given that the current evidence is unclear, additional, well-designed, longitudinal cohort studies examining sleep and cancer are warranted. Future work could uncover the types of cancer that are most closely associated with sleep disturbances, as well as the dose-response relationship for a given sleep-related risk factor. Epidemiological studies are uniquely positioned to evaluate population level relationships that may otherwise go undetected in the laboratory.

### Management of Sleep Disruption in Patients

The current approach to managing sleep disturbances in patients with cancer initially focuses on non-pharmacologic treatments, such as sleep hygiene modifications, cognitive behavioral therapy for insomnia (CBTI), and relaxation techniques. CBTI is an evidence-based, structured program that is used to combat insomnia in the general population. Recent trials have found that CBT-I can significantly improve sleep measures in breast cancer survivors and patients undergoing treatment (Savard et al., 2005; Espie et al., 2008; Berger et al., 2009). Results from these studies suggest that cancer patients may benefit from psychological interventions. If these are not available or successful, pharmacologic treatments that have not been thoroughly studied in cancer populations are usually prescribed, such as benzodiazepine receptor agonists (e.g., zolpidem) and benzodiazepines (lorazepam). In a recent survey of cancer patients, 22.6% were taking medication for sleep problems, with half of these patients using the medication every day for longer than 6 months. Long term use of hypnotic medications appears to be widely used by patients with cancer despite limited data regarding long-term efficacy and possible adverse effects, such as daytime sedation and cognitive impairments. Furthermore, chronic use is associated with dependence and offers no real evidence of benefit (Kripke, 2000). The current approach to sleep problems in patients with cancer is not sufficient given the impact sleep disruption has on both quality of life and cancer outcomes. The etiology of cancer induced sleep disruption is likely different than that of the general population, so standard sleep treatments may not offer the same benefit to patients with cancer. Recent advances in fMRI technology and analysis have allowed researchers to accurately screen for vulnerability to sleep deprivation using resting-state network measures coupled with machine learning (Xu et al., 2021). This type of novel screening tool may have useful applications in the context of cancer wherein patients could be assessed for their risk of cancer-induced sleep disruption during the initial stages of their diagnosis and treatment.

### Melatonin as a Treatment

Melatonin is a physiological signal of darkness that is associated with sleep in humans. The role of melatonin suppression has been causally investigated as a contributor to increased cancer risk. Thus, considerable research has focused on the role of artificial light exposure during the night and melatonin suppression, which appears to be related to an increased cancer risk (Yang et al., 2014; Jardim-Perassi et al., 2014). *In vivo* studies have demonstrated that melatonin treatment reduced tumor size and cell proliferation, as well as a decrease in VEGF receptor 2 density, in mice with breast cancer xenografts (Jardim-Perassi et al., 2014). Moreover, important to tumor suppression, melatonin has gained increased notoriety over the last several decades for its antioxidant properties as melatonin influences both antioxidant enzyme activity and cellular mRNA levels for these enzymes (Tan et al., 1993; Steinhilber et al., 1995; Reiter, 1998; Reiter and Maestroni, 1999).

Experimental models and epidemiological studies indicate that melatonin may have an onco-protective role. These studies have shown that melatonin improves the sensitivity of cancers to chemotherapy and has the potential to reverse drug resistance in tumors (Uguz et al., 2012; Dauchy et al., 2014; Xiang et al., 2015). Moreover, melatonin has been shown to inhibit molecular processes associated with metastasis (Su et al., 2017). Prior to cancer initiation, melatonin also serves as a free radical scavenger (Tan et al., 2002), preventing DNA damage that could lead to oncogenic mutations. Given these findings, a more complete understanding of melatonin in homeostasis and malignancy would be of immediate clinical utility. Melatonin is synthesized by the pineal gland and represents a biological timing signal that is driven by the activity of the SCN of the anterior hypothalamus and synchronized to the light/dark cycle (Claustrat et al., 2005). Experimental studies examining how changes in this neurocircuitry affect oncogenesis, cancer progression, and cancer treatment would be highly valuable to the clinic. For example, bilateral electrolytic lesion of the SCN results in disruption of circadian rhythms and subsequent acceleration of tumor growth (Filipski et al., 2002). Treatment modalities targeting this neurocircuitry or melatonin itself could prove useful in cancer prevention or as an adjuvant of current cancer therapies. The information gathered from these studies should be translated to the clinic, granting providers the knowledge to deliver evidence-based cancer prevention recommendations.

In clinical trials where melatonin was used as an adjuvant therapy with other chemotherapeutic drugs there was enhanced therapeutic efficacy, higher survival rate, and increases sleep and quality of life (Cerea et al., 2003; Lissoni et al., 2003). Support for the anti-cancer properties of melatonin supplementation is less consistent in epidemiological studies. Several studies report a protective role of melatonin in cancer (Basler et al., 2014), while other studies find no significant relationship (Travis et al., 2004; Sturgeon et al., 2014). Thus, melatonin treatment may help resolve the sleep disturbances experienced by patients with cancer while also decreasing tumor progression. However, melatonin is “messy” in that it signals through two distinct receptors on majority of cells and has many receptor-independent effects which could decrease the efficacy of melatonin as a therapeutic strategy in cancer patients.

## Unanswered Questions and Forward Directions for the Field

### Employing Modern Neuroscience Techniques to Address These Questions

We are just beginning to appreciate the bi-directional communication between cancer and the nervous system. The last decade has seen an explosion in discoveries regarding the role of nerves in the tumor microenvironment. Recent advances in modern neuroscience techniques have allowed us to expand upon these findings and trace their influence on the central nervous system. These modern techniques (e.g., optogenetics, calcium imaging) should be leveraged to dissect the neural circuits disrupted by cancer, leading to a more complete understanding of the pathophysiology of malignancy. Uncovering the neural correlates of cancer-related sleep disruption has the potential to inform novel treatment approaches and screening tools that will improve patient care and oncology outcomes. Despite recent progress, many questions remain regarding sleep in the context of cancer.

A potential focus for preclinical discovery will be to localize specific sleep-associated brain nuclei deregulated by cancer and then employ targeted stimulation or inhibition of these circuits to see what their role is in the development of cancer-associated sleep disruption. Advances in neuroimaging, such as serial two photon tomography (STPT), are already allowing researchers to examine how an entire brain responds to selective perturbations (e.g., drug administration, social interaction) (Kim et al., 2015; Ueda et al., 2020). Unbiased screens of cancer-induced changes in neuronal activity will allow for identification and subsequent manipulation of specific neuronal circuits. Neuroendocrine and metabolic changes must also be elucidated as they are closely tied to sleep physiology. Central nervous system sensitivity to immune related changes is another area that will inform many unanswered questions.

A detailed understanding of how cytokines and metabolic factors affect the central nervous system will become increasingly more important as future cancer treatments targeting or harnessing the immune system become more widely used. Immune checkpoint inhibitors, such as antibodies targeting PD-1/PD1 or CLTA-4, and cellular therapy utilizing chimeric antigen receptor (CAR) T cells are all likely to alter the cancer-induced cytokine and metabolic milieu. Developing a neuroimmune effector map now will translate into the ability to predict the effects targeted immunotherapies may have on the CNS.

Computational approaches will be key in handling the large amounts of data generated by EEG/polysomnography and pulling out important cancer-related changes in brain function. These include machine learning/artificial intelligence techniques, which can (in certain situations) identify small but important changes in data structure that may be missed by classic sleep scoring techniques. This will promote a research cycle where computational approaches allow us to generate hypotheses from large datasets that can then be tested and refined *in vivo*. Finally, we need additional input from oncologists, as not all cancers influence sleep in the same way, and the amelioration of these problems will likely require close collaboration with those who know the details of specific cancer-secreted molecules, propensity for neural invasion, and treatment resistance.

## Author Contributions

AB prepared figures and figure legends. JB supervised the work. All authors designed, wrote, and edited the manuscript.

## Conflict of Interest

The authors declare that the research was conducted in the absence of any commercial or financial relationships that could be construed as a potential conflict of interest.

## Publisher’s Note

All claims expressed in this article are solely those of the authors and do not necessarily represent those of their affiliated organizations, or those of the publisher, the editors and the reviewers. Any product that may be evaluated in this article, or claim that may be made by its manufacturer, is not guaranteed or endorsed by the publisher.
